# Supplementary material for: A multi-ethnic proteomic profiling analysis in Alzheimer’s disease identifies the disparities in dysregulation of proteins and pathogenesis
Source: PeerJ. 2024 Jul 18;12:e17643. doi: 10.7717/peerj.17643 (PMC11260413; doi:10.7717/peerj.17643)
Supplement: Supplemental Information 1 [file peerj-12-17643-s001.docx]

**Supplementary Table 1**

Significant KEGG pathways and GO terms enriched for CADvC and MADvC.

| Module | GO ID | Term Name | Adjusted p-value | Associated Proteins |
| --- | --- | --- | --- | --- |
| CADvC | GO:0031091 | platelet alpha granule | 0.000 | A1BG, AHSG, SELENOP |
|  | GO:0043312 | neutrophil degranulation | 0.002 | A1BG, AHSG, KRT1, LTF |
|  | GO:0002252 | immune effector process | 0.005 | A1BG, AHSG, KRT1, LTF, RBP4 |
|  | GO:0001775 | cell activation | 0.010 | A1BG, AHSG, KRT1, LTF, SELENOP |
|  | GO:0002576 | platelet degranulation | 0.000 | A1BG, AHSG, ECM1, SELENOP, TF |
|  | GO:0072562 | blood microparticle | 0.000 | A1BG, AHSG, VDBP, ITIH1, KRT1, TF |
|  | GO:0062023 | collagen-containing extracellular matrix | 0.000 | A1BG, AHSG, ECM1, F12, F9, ITIH1, KRT1 |
|  | GO:0030141 | secretory granule | 0.000 | A1BG, AHSG, ECM1, KRT1, LTF, SELENOP, TF |
|  | GO:0045055 | regulated exocytosis | 0.000 |  |
|  | GO:0051649 | establishment of localization in cell | 0.001 | A1BG, AHSG, ECM1, F9, KRT1, LTF, SELENOP, TF |
|  | GO:0016192 | vesicle-mediated transport | 0.000 |  |
|  | GO:0032940 | secretion by cell | 0.000 | A1BG, AHSG, ECM1, KRT1, LTF, RBP4, SELENOP, TF |
|  | GO:0070013 | intracellular organelle lumen | 0.015 | A1BG, AHSG, ECM1, F9, VDBP, KRT1, LTF, SELENOP, TF |
|  | GO:0002376 | immune system process | 0.001 | A1BG, AHSG, ECM1, F12, KRT1, LTF, RBP4, SELENOP, TF |
|  | GO:0006810 | transport | 0.003 | A1BG, AHSG, ECM1, F9, VDBP, KRT1, LTF, RBP4, SELENOP, TF |
|  | GO:0012505 | endomembrane system | 0.002 | A1BG, AHSG, ECM1, F12, F9, HGFAC, KRT1, LTF, SELENOP, TF |
|  | GO:0071944 | cell periphery | 0.002 | A1BG, AHSG, ECM1, F12, F9, ITIH1, KRT1, KRT10, LTF, SELENOP, TF |
|  | GO:0005615 | extracellular space | 0.000 | A1BG, AHSG, ECM1, F12, F9, VDBP, HGFAC, ITIH1, KRT1, KRT10, LTF, RBP4, SELENOP, TF |
|  | GO:0043227 | membrane-bounded organelle | 0.017 |  |
|  | GO:0005788 | endoplasmic reticulum lumen | 0.005 | AHSG, F9, TF |
|  | GO:0004866 | endopeptidase inhibitor activity | 0.002 | AHSG, ITIH1, LTF |
|  | GO:0030500 | regulation of bone mineralization | 0.000 | AHSG, ECM1, LTF |
|  | GO:0030282 | bone mineralization | 0.000 |  |
|  | GO:0032101 | regulation of response to external stimulus | 0.021 | AHSG, F12, KRT1, LTF |
|  | GO:0010466 | negative regulation of peptidase activity | 0.000 | AHSG, ECM1, ITIH1, LTF |
|  | GO:0001501 | skeletal system development | 0.002 | AHSG, ECM1, LTF, RBP4 |
|  | GO:0051093 | negative regulation of developmental process | 0.009 |  |
|  | GO:0005783 | endoplasmic reticulum | 0.023 | AHSG, F12, F9, HGFAC, TF |
|  | GO:0050790 | regulation of catalytic activity | 0.050 | AHSG, ECM1, ITIH1, LTF, TF |
|  | GO:0048585 | negative regulation of response to stimulus | 0.017 | AHSG, ECM1, F12, KRT1, LTF |
|  | GO:0006954 | inflammatory response | 0.002 | AHSG, ECM1, F12, KRT1, SELENOP |
|  | GO:0009605 | response to external stimulus | 0.035 | AHSG, F12, KRT1, LTF, SELENOP, TF |
|  | GO:0009888 | tissue development | 0.006 | AHSG, ECM1, KRT1, KRT10, LTF, RBP4 |
|  | GO:0050793 | regulation of developmental process | 0.014 |  |
|  | GO:0051239 | regulation of multicellular organismal process | 0.005 | AHSG, ECM1, F12, KRT1, LTF, RBP4, TF |
|  | GO:0006952 | defense response | 0.001 | AHSG, ECM1, F12, KRT1, LTF, SELENOP, TF |
|  | GO:0048583 | regulation of response to stimulus | 0.044 |  |
|  | GO:0140096 | catalytic activity, acting on a protein | 0.003 | AHSG, ECM1, F12, F9, HGFAC, ITIH1, LTF, TF |
|  | GO:0006950 | response to stress | 0.003 | AHSG, ECM1, F12, F9, HGFAC, KRT1, LTF, SELENOP, TF |
|  | GO:0048513 | animal organ development | 0.001 | AHSG, ECM1, VDBP, KRT1, KRT10, LTF, RBP4, SELENOP, TF |
|  | GO:0019538 | protein metabolic process | 0.005 | AHSG, ECM1, F12, F9, HGFAC, ITIH1, KRT1, KRT10, LTF, TF |
|  | GO:0043170 | macromolecule metabolic process | 0.015 | AHSG, ECM1, F12, F9, VDBP, HGFAC, ITIH1, KRT1, KRT10, LTF, RBP4, TF |
|  | GO:0032501 | multicellular organismal process | 0.003 | AHSG, ECM1, F12, F9, VDBP, HGFAC, KRT1, KRT10, LTF, RBP4, SELENOP, TF |
|  | GO:0005198 | structural molecule activity | 0.027 | ECM1, KRT1, KRT10 |
|  | GO:1902533 | positive regulation of intracellular signal transduction | 0.015 | ECM1, LTF, SELENOP, TF |
|  | GO:0002682 | regulation of immune system process | 0.015 | ECM1, KRT1, LTF, RBP4, SELENOP |
|  | GO:0031638 | zymogen activation | 0.000 | F12, F9, HGFAC |
|  | GO:0005509 | calcium ion binding | 0.005 | F12, F9, ITIH1, SELENOP |
|  | GO:0098542 | defense response to other organism | 0.005 | F12, KRT1, LTF, SELENOP, TF |
|  | GO:0007596 | blood coagulation | 0.000 | F12, F9, HGFAC, KRT1, SELENOP |
|  | GO:0065008 | regulation of biological quality | 0.009 | F12, F9, HGFAC, KRT1, LTF, RBP4, SELENOP, TF |
|  | GO:0008289 | lipid binding | 0.007 | VDBP, LTF, RBP4, SELENOP |
|  | GO:0006959 | humoral immune response | 0.008 | KRT1, LTF, TF |
|  | GO:0048878 | chemical homeostasis | 0.021 | KRT1, LTF, RBP4, TF |
|  | GO:0001894 | tissue homeostasis | 0.000 |  |
|  | GO:0009986 | cell surface | 0.009 | KRT10, LTF, SELENOP, TF |
|  | GO:0042742 | defense response to bacterium | 0.007 | LTF, SELENOP, TF |
| MADvC | GO:0051241 | negative regulation of multicellular organismal process | 0.005 | APOA4, F12, FGA |
|  | GO:0051336 | regulation of hydrolase activity | 0.006 | APOA4, C4A, ITIH4 |
|  | GO:0031410 | cytoplasmic vesicle | 0.044 | APOA4, FGA, ITIH4 |
|  | GO:0046872 | metal ion binding | 0.036 | APOA4, C2, F12, FGA |
|  | GO:0051049 | regulation of transport | 0.003 | APOA4, C2, C4A, FGA |
|  | GO:0062023 | collagen-containing extracellular matrix | 0.000 | APOA4, F12, FGA, ITIH4 |
|  | GO:0072562 | blood microparticle | 0.000 | APOA4, C4A, FGA, ITIH4 |
|  | GO:0045087 | innate immune response | 0.000 | APOA4, C2, C4A, F12, FGA |
|  | GO:0016787 | hydrolase activity | 0.003 | APOA4, C2, C4A, F12, ITIH4 |
|  | GO:0006810 | transport | 0.010 | APOA4, C2, C4A, FGA, ITIH4 |
|  | GO:0006952 | defense response | 0.000 | APOA4, C2, C4A, F12, FGA, ITIH4 |
|  | GO:0019538 | protein metabolic process | 0.002 |  |
|  | GO:0070062 | extracellular exosome | 0.000 |  |
|  | KEGG:05171 | Coronavirus disease | 0.000 | C2, C4A, FGA |
|  | GO:0002250 | adaptive immune response | 0.003 |  |
|  | GO:0006959 | humoral immune response | 0.001 |  |
|  | KEGG:04610 | Complement and coagulation cascades | 0.000 | C2, C4A, F12, FGA |
|  | GO:0004175 | endopeptidase activity | 0.000 | C2, C4A, F12, ITIH4 |
|  | GO:0006954 | inflammatory response | 0.004 | C4A, F12, ITIH4 |
|  | GO:0051248 | negative regulation of protein metabolic process | 0.006 |  |
|  | GO:0030162 | regulation of proteolysis | 0.003 |  |
