# Supplementary material for: A multi-ethnic proteomic profiling analysis in Alzheimer’s disease identifies the disparities in dysregulation of proteins and pathogenesis
Source: PeerJ. 2024 Jul 18;12:e17643. doi: 10.7717/peerj.17643 (PMC11260413; doi:10.7717/peerj.17643)
Supplement: Supplemental Information 2 — Note: Blue dot (Hedges’ g) represents the SMD for each individual study where the positive value indicates higher levels of proteins in AD compared to normal controls. Green dot indicates the total SMD controlled by the combined effects of prediction interval (PI) and confidence interval (CI). Total SMD with positive value indicates upregulation of the proteins in AD group. [file peerj-12-17643-s002.docx]

Supplementary Table 1: Forest plots for abundance levels of the dysregulated proteins identified in CADvC among groups of AD patient vs normal control.

| Protein/ Study | Alzheimer's Disease | | | Control | | | Weight (%) | Std. Mean Difference (Random) [95% CI] |  |
| --- | --- | --- | --- | --- | --- | --- | --- | --- | --- |
|  | Mean | SD | No. sample | Mean | SD | No. sample |  |  |  |
| **Vitamin D-binding protein (*VDBP*)** | | | | | | | | | 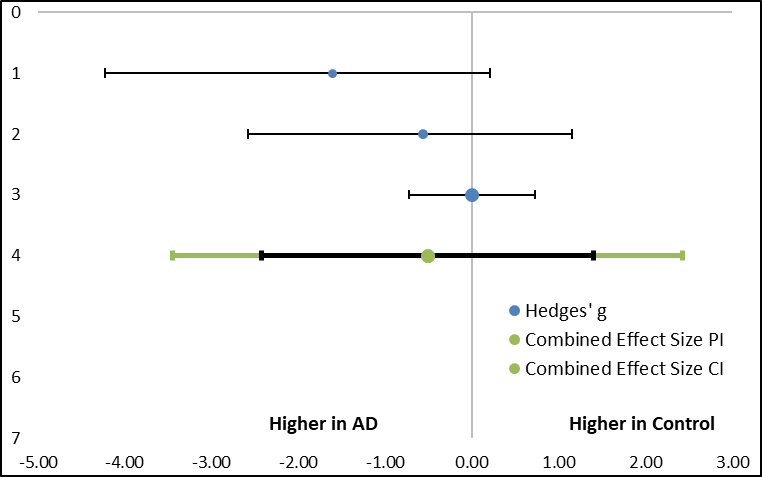 |
| Current study | 50558.53 | 8372.76 | 3 | 32948.93 | 9094.2 | 3 | 21.94 | -1.61[-4.25, 0.21] |  |
| Eldem et al. (2022) | 115166667 | 155985843 | 3 | 34413333 | 38369030.9 | 3 | 27.68 | -0.57[-2.58, 1.15] |  |
| Nielsen et al. (2021) | 3265070 | 2364509 | 20 | 4041308.3 | 2341750 | 12 | 50.38 | 0.00[-0.73, 0.73] |  |
|  |  |  |  |  |  |  |  |  |  |
| Total (95% CI) |  |  | 26 |  |  | 18 | 100 | -0.51[-2.42, 1.41] |  |
| Heterogeneity: Q= 3.53, T2= 0.27, I2= 43.42%, df=1(P=0.44) | | | | | |  | | |  |
| Pearson Correlation (r)= 0.3 | | | | | |  | | |  |
| Test for overall effect: Z= -1.15(P=0.17) | | | | | |  | | |  |
| **Serotransferrin (*TF*)** | | | | | | | | |  |
| Current study | 282519.7 | 37640.99 | 3 | 205149 | 4237.47 | 3 | 6.58 | -2.30[-5.47, -0.30] |  |
| Eldem et al. (2022) | 231700000 | 317280901 | 3 | 984333333 | 1296940374 | 3 | 10.7 | 0.63[-1.08, 2.68] |  |
| Kononikhin et al. (2022) | 18394.1 | 3732.68 | 96 | 19112.33 | 2639.2 | 86 | 32.04 | 0.22[-0.07, 0.51] |  |
| Nielsen et al. (2021) | 35757100 | 20550497 | 20 | 30780083 | 19399142 | 12 | 21.6 | -0.24[-0.98, 0.48] | 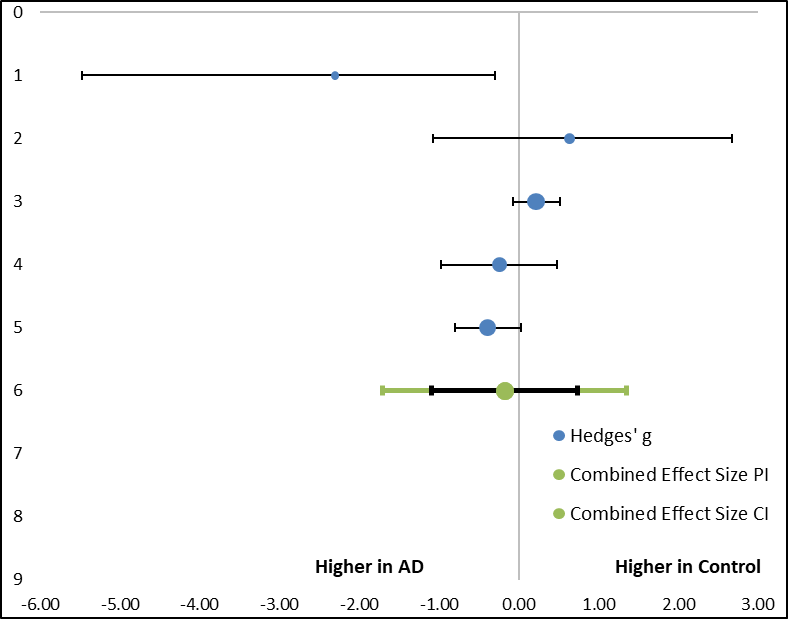 |
| Ashraf et al. (2020) | 0.003 | 0.017 | 25 | 0 | 0.006 | 238 | 29.08 | -0.39[-0.80, 0.02] |  |
|  |  |  |  |  |  |  |  |  |  |
| Total (95% CI) |  |  | 147 |  |  | 342 | 100 | -0.18[-1.09, 0.73] |  |
| Heterogeneity: Q= 13.01, T2= 0.2, I2= 69.26%, df=1(P=0.85) | | | | | |  | | |  |
| Pearson Correlation (r)= 0.09 | | | | | |  | | |  |
| Test for overall effect: Z= -0.54(P=0.01) | | | | |  |  | | |  |
| **Lactotransferrin (*LTF*)** | | | | | | | | |  |
| Current study | 282519.7 | 37640.99 | 3 | 205149 | 4237.47 | 3 | 6.58 | -2.30[-5.47, -0.30] |  |
| Eldem et al. (2022) | 231700000 | 317280901 | 3 | 984333333 | 1296940374 | 3 | 10.7 | 0.63[-1.08, 2.68] |  |
| Kononikhin et al. (2022) | 18394.1 | 3732.68 | 96 | 19112.33 | 2639.2 | 86 | 32.04 | 0.22[-0.07, 0.51] | 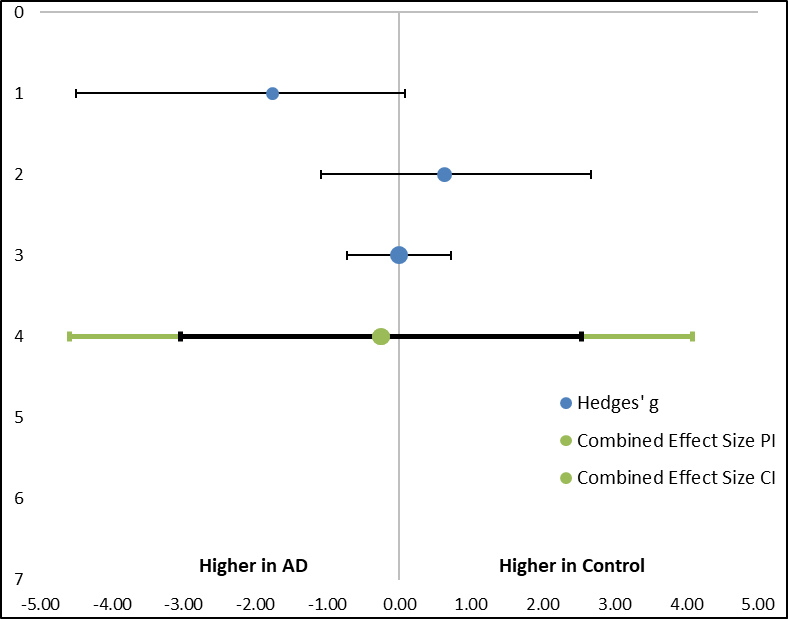 |
| Nielsen et al. (2021) | 35757100 | 20550497 | 20 | 30780083 | 19399142 | 12 | 21.6 | -0.24[-0.98, 0.48] |  |
| Ashraf et al. (2020) | 0.003 | 0.017 | 25 | 0 | 0.006 | 238 | 29.08 | -0.39[-0.80, 0.02] |  |
|  |  |  |  |  |  |  |  |  |  |
| Total (95% CI) |  |  | 147 |  |  | 342 | 100 | -0.18[-1.09, 0.73] |  |
| Heterogeneity: Q= 5.31, T2= 0.6, I2= 62.32%, df=1(P=0.95) | | | | |  |  |  |  |  |
| Pearson Correlation (r)= 0.15 | | | | |  |  |  |  |  |
| Test for overall effect: Z= -0.39(P=0.07) | | | | |  |  |  |  |  |

| **Alpha-2-HS-glycoprotein (*AHSG*)** | | | | |  |  | | |  |
| --- | --- | --- | --- | --- | --- | --- | --- | --- | --- |
| Current study | 36233.53 | 5362.53 | 3 | 28093.5 | 3976.72 | 3 | 10 | -1.38[-3.84, 0.39] |  |
| Eldem et al. (2022) | 32603000 | 53284313 | 3 | 71563333 | 109501357 | 3 | 11.75 | 0.36[-1.38, 2.28] |  |
| Kononikhin et al. (2022) | 2488.85 | 354.95 | 96 | 2569.43 | 420.7 | 86 | 23.14 | 0.21[-0.08, 0.5] |  |
| Nielsen et al. (2021) | 3692498 | 3234214.7 | 20 | 2377034.2 | 2181779.4 | 12 | 18.03 | -1.01[-1.81, 0.27] | 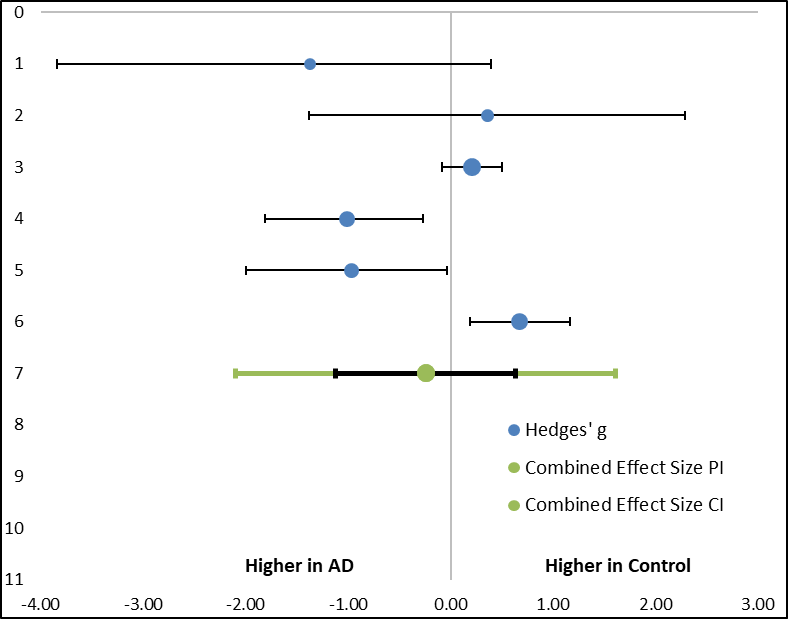 |
| Kitamura et al. (2017) | 1.18 | 0.42 | 9 | 0.83 | 0.26 | 10 | 15.87 | -0.97[-2, 0.03] |  |
| Smith et al. (2011) | 0.261 | 0.059 | 34 | 0.299 | 0.053 | 34 | 21.21 | 0.67[0.19,1.17] |  |
|  |  |  |  |  |  |  |  |  |  |
| Total (95% CI) |  |  | 131 |  |  | 114 | 100 | -0.24[-1.12, 0.64] |  |
|  |  |  |  |  |  |  |  |  |  |
| Heterogeneity: Q= 23.73, T2= 0.4, I2= 79%, df=1(P=0.74) | | | | |  |  | | |  |
| Pearson Correlation (r)= 0.13 | | | | |  |  | | |  |
| Test for overall effect: Z= -0.71(P<0.001) | | | | |  |  | | |  |
| **Coagulation factor IX (*F9*)** | | |  | |  |  | | |  |
| Current study | 273.33 | 33.64 | 3 | 221.23 | 36.57 | 3 | 21.12 | -1.18[-3.52, 0.56] |  |
| Nielsen et al. (2021) | 37695.89 | 42814.29 | 20 | 0 | 0 | 12 | 78.88 | 1.08[-1.88, -0.33] |  |
|  |  |  |  |  |  |  |  |  |  |
| Total (95% CI) |  |  | 23 |  |  | 15 | 100 | -1.1[-1.64, -0.56] | 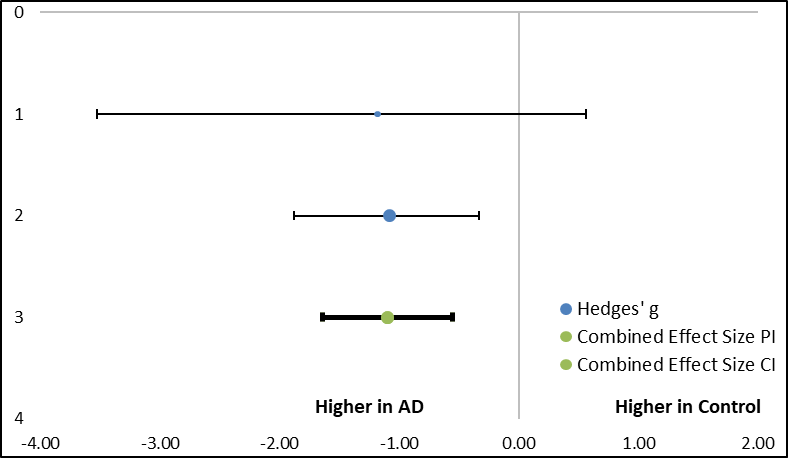 |
| Heterogeneity: Q=0.02, T2= 0, I2= 0, df=1(P=0.9) | | | | |  |  | | |  |
| Pearson Correlation (r)= 0.5 | | | | |  |  | | |  |
| Test for overall effect: Z= -25.75(P=0.9) | | | | |  |  | | |  |
| **Selenoprotein P (*SELENOP)*** | | |  | |  |  | | |  |
| Current study | 2500.93 | 455.12 | 3 | 1954.97 | 278.07 | 3 | 20.3 | -1.16[-3.48, 0.58] |  |
| Nielsen et al. (2021) | 56729.25 | 34538.62 | 20 | 26371.58 | 41964.91 | 12 | 79.7 | 0.79[-1.57, -0.06] |  |
|  |  |  |  |  |  |  |  |  | 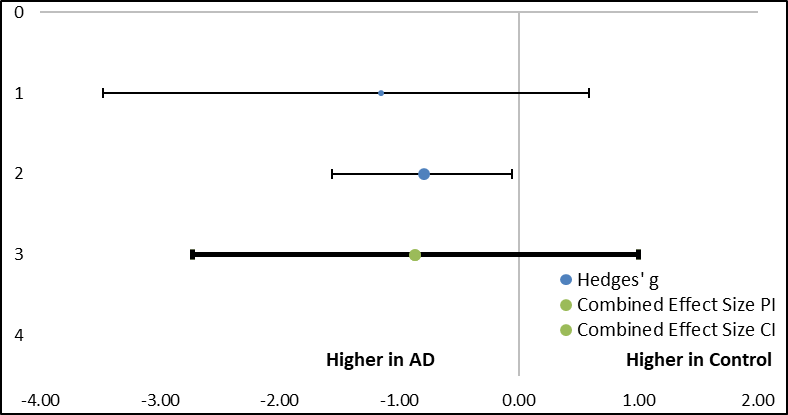 |
| Total (95% CI) |  |  | 23 |  |  | 15 | 100 | -0.86[-2.73, 1] |  |
| Heterogeneity: Q=0.2, T2= 0, I2= 0, df=1(P=0.66) | | | | |  |  | | |  |
| Pearson Correlation (r)= 0.41 | | | | |  |  | | |  |
| Test for overall effect: Z= 5.9(P=0.66) | | | | |  |  | |  |  |
| **Retinol binding protein 4 (*RBP4)*** | | | | |  |  | | |  |
| Current study | 17376.97 | 4719.76 | 3 | 10155.53 | 3262.62 | 3 | 21.45 | -1.42[-3.92, 0.36] |  |
| Kononikhin et al. (2022) | 671.27 | 199.35 | 96 | 654.54 | 162.09 | 86 | 43.32 | 0.09[-0.38, 0.2] |  |
| Nielsen et al. (2021) | 190306.5 | 177495.97 | 20 | 10206.75 | 35356.9 | 12 | 35.23 | -1.23[-2.05, -0.47] |  |
|  |  |  |  |  |  |  |  |  | 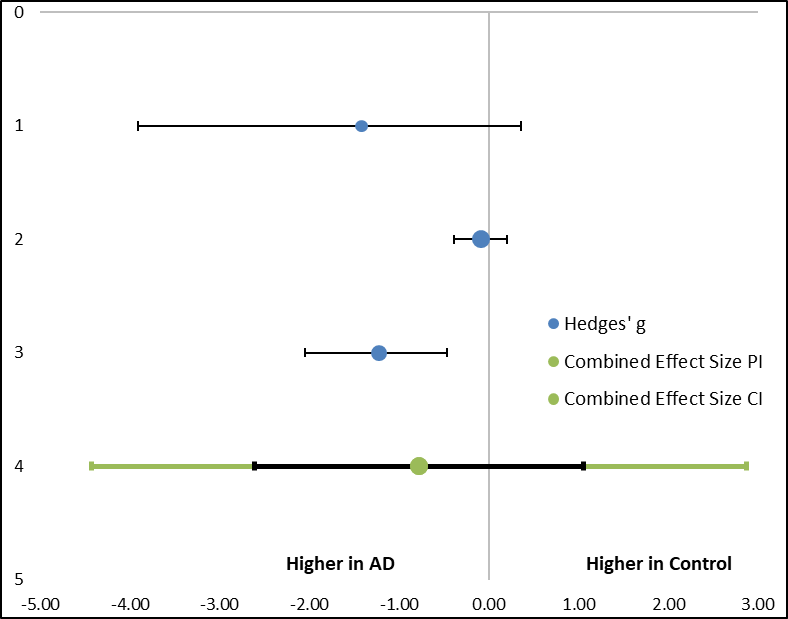 |
| Total (95% CI) |  |  | 119 |  |  | 101 | 100 | -0.78[-2.61, 1.05] |  |
| Heterogeneity: Q= 9.81, T2= 0.54, I2= 79.61%, df=1(P=0.13) | | | | |  |  | | |  |
| Pearson Correlation (r)= 0.39 | | | | |  |  | | |  |
| Test for overall effect: Z= -1.82(P=0.007) | | | | |  |  | | |  |
| **Extracellular matrix protein 1 (*ECM1)*** | | | | |  |  | | |  |
| Current study | 4260.6 | 1133.65 | 3 | 2929.27 | 655.67 | 3 | 25.8 | -1.15[-3.47, 0.59] |  |
| Eldem et al. (2022) | 536000 | 578954.23 | 3 | 3396666.667 | 3496188.4 | 3 | 27.01 | 0.91[-0.81, 3.09] |  |
| Nielsen et al. (2021) | 139317.5 | 175240.76 | 20 | 1314248 | 3054602.26 | 12 | 47.19 | 0.00[-0.73, 0.73] |  |
|  |  |  |  |  |  |  |  |  |  |
| Total (95% CI) |  |  | 26 |  |  | 18 | 100 | -0.05[-2.33, 2.23] | 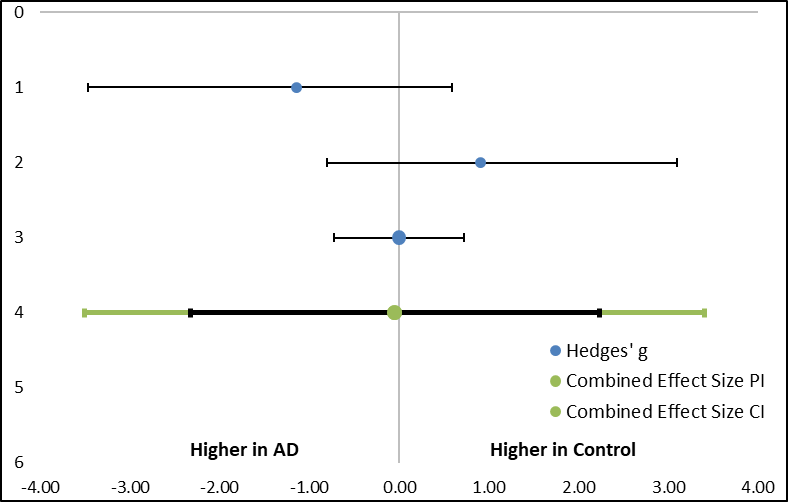 |
| Heterogeneity: Q= 4.14, T2= 0.36, I2= 51.66%, df=1(P=0.8) | | | | |  |  | | |  |
| Pearson Correlation (r)= 0.03 | | | | |  |  | | |  |
| Test for overall effect: Z= -0.09(P=0.12) | | | | |  |  | | |  |
| **Inter-alpha-trypsin inhibitor heavy chain H1 (*ITIH1*)** | | | | |  |  | | |  |
| Current study | 802.76 | 168.21 | 3 | 1798.53 | 769.02 | 3 | 21.31 | 1.43[-0.35, 3.93] |  |
| Eldem et al. (2022) | 653666.667 | 795927.34 | 3 | 37666666.67 | 65240580.4 | 3 | 25.77 | 0.64[-1.08, 2.68] | 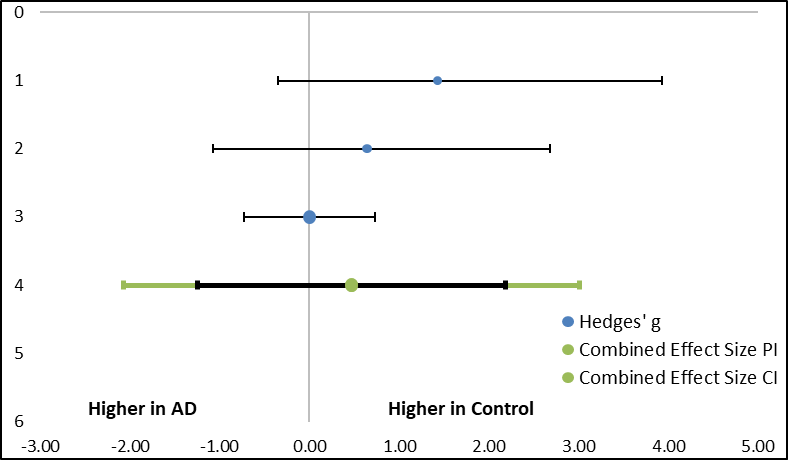 |
| Nielsen et al. (2021) | 1353494.5 | 607877.47 | 20 | 1314248 | 3054602.26 | 12 | 52.92 | 0.00[-0.73, 0.73] |  |
|  |  |  |  |  |  |  |  |  |  |
| Total (95% CI) |  |  | 26 |  |  | 18 | 100 | -0.47[-1.25, 2.19] |  |
| Heterogeneity: Q= 3.1, T2= 0.19, I2= 35.39%, df=1(P=0.27) | | | | |  |  | | |  |
| Pearson Correlation (r)= 0.27 | | | | |  |  | | |  |
| Test for overall effect: Z= 1.17(P=0.21) | | | | |  |  | | |  |
| **Alpha-1B-glycoprotein (*A1BG*)** | | | | |  |  | | |  |
| Current study | 13621.47 | 165.01 | 3 | 10474.6 | 1441.18 | 3 | 12.11 | -2.44[-5.73, -0.4] |  |
| Eldem et al. (2022) | 15600000 | 21494243 | 3 | 67713333.33 | 100822966 | 3 | 18.88 | 0.57[-1.15, 2.58] |  |
| Kononikhin et al. (2022) | 1673.22 | 305.59 | 96 | 1539.71 | 426.12 | 86 | 38.44 | -0.36[-0.66, -0.07] |  |
| Nielsen et al. (2021) | 1018100 | 935679.6 | 20 | 314711.17 | 340138.3 | 12 | 30.57 | 0.38[-0.34, 1.12] | 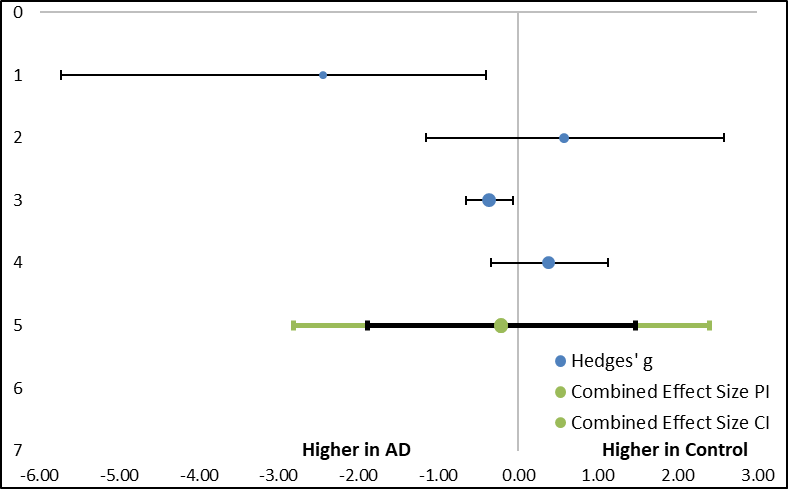 |
|  |  |  |  |  |  |  |  |  |  |
| Total (95% CI) |  |  | 122 |  |  | 104 | 100 | -0.21[-1.89, 1.47] |  |
| Heterogeneity: Q= 10.35, T2= 0.39, I2= 71.02%, df=1(P=0.84) | | | | |  |  | |  |  |
| Pearson Correlation (r)= 0.11 | | | | |  |  | |  |  |
| Test for overall effect: Z= -0.4(P=0.01) | | | | |  |  | | |  |
| **type II cytoskeletal 1 (*KRT1*)** | | | | |  |  | | |  |
| Current study | 6560.57 | 1408.41 | 3 | 4258.5 | 543.54 | 3 | 26.58 | -1.72[-4.43, 0.12] |  |
| Eldem et al. (2022) | 1366666667 | 957200780 | 3 | 151033333.3 | 120453324 | 3 | 28.66 | -1.33[-3.78, 0.42] |  |
| Nielsen et al. (2021) | 59974400 | 31079065 | 20 | 60168666.67 | 51781784 | 12 | 44.76 | 0.00[-0.73, 0.73] |  |
|  |  |  |  |  |  |  |  |  | 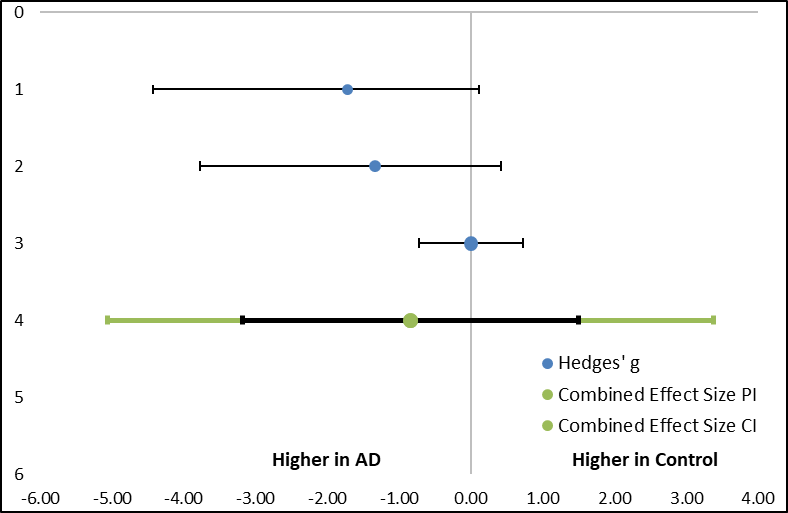 |
| Total (95% CI) |  |  | 26 |  |  | 18 | 100 | -0.84[-3.18, 1.50] |  |
| Heterogeneity: Q= 5.38, T2= 0.67, I2= 62.83%, df=1(P=0.03) | | | | |  |  | | |  |
| Pearson Correlation (r)= 0.46 | | | | |  |  | | |  |
| Test for overall effect: Z= -1.54(P=0.07) | | | | |  |  | | |  |

| **type II cytoskeletal 1 (*KRT10*)** | |  |  | |  |  | | |  |
| --- | --- | --- | --- | --- | --- | --- | --- | --- | --- |
| Current study | 3056.37 | 728.98 | 3 | 2018.33 | 318.95 | 3 | 26.99 | -1.47[-4., 0.31] |  |
| Eldem et al. (2022) | 1366666667 | 957200780.1 | 3 | 151033333.3 | 120453324.3 | 3 | 27.27 | -1.42[-3.92, 0.36] |  |
| Nielsen et al. (2021) | 16568060 | 10707616.77 | 20 | 26449475 | 26366345.73 | 12 | 45.74 | 0.00[-0.73, 0.73] |  |
|  |  |  |  |  |  |  |  |  | 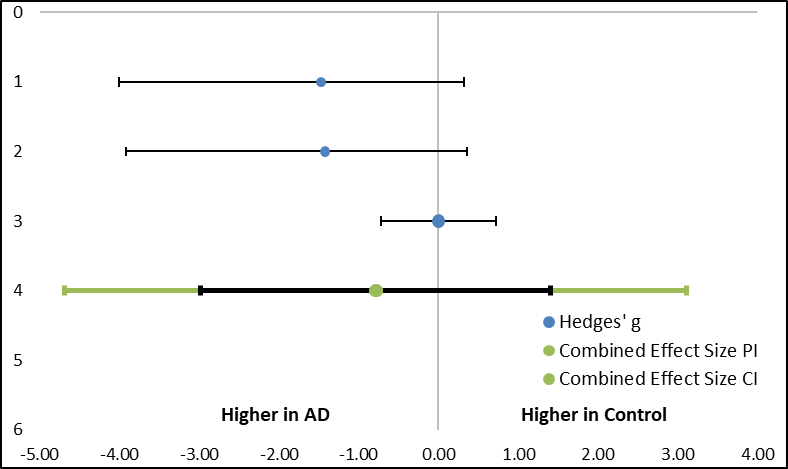 |
| Total (95% CI) |  |  | 26 |  |  | 18 | 100 | -0.78[-2.98, 1.41] |  |
| Heterogeneity: Q= 4.9, T2= 0.56, I2= 59.3%, df=1(P=0.03) | | | | |  |  | | |  |
| Pearson Correlation (r)=0.44 | | | | |  |  | | |  |
| Test for overall effect: Z= -1.54(P=0.08) | | | | |  |  | | |  |

Note: Blue dot (Hedges’ g) represents the SMD for each individual study where the positive value indicates higher levels of proteins in AD compared to normal controls. Green dot indicates the total SMD controlled by the combined effects of prediction interval (PI) and confidence interval (CI). Total SMD with positive value indicates upregulation of the proteins in AD group.
