# Supplementary material for: A multi-ethnic proteomic profiling analysis in Alzheimer’s disease identifies the disparities in dysregulation of proteins and pathogenesis
Source: PeerJ. 2024 Jul 18;12:e17643. doi: 10.7717/peerj.17643 (PMC11260413; doi:10.7717/peerj.17643)
Supplement: Supplemental Information 4 [file peerj-12-17643-s004.docx]

Table S4: List of identified proteins in CADvC and their role and functions related to AD.

| Protein | Role and functions related to AD |
| --- | --- |
| Vitamin D-binding protein (*VDBP*) | - A multifunctional protein which found to increase levels in plasma and CSF of AD patients (Moon et al. 2013). - Acts as an *Aβ* scavenger that attenuated the aggregation and accumulation of *Aβ*, suppressing the Aβ-mediated AD pathology (Bishnoi et al. 2015; Jeon et al. 2019). |
| Serotransferrin (*TF*) | - The dysfunction of protein *TF* will lead to iron overload in the brain which might cause neurodegeneration (Ficiarà et al. 2021). - The decline in the level of *TF* caused the propagation of Aβ-toxicity in the brain, subsequently deteriorating AD progression (Ashraf et al. 2020; Guan et al. 2020). - *TF* was evidenced to associate with the elevation of AD risk among the Chinese population (Zhang et al. 2003) which was again confirmed in our findings. |
| Lactotransferrin (*LTF*) | - Modulate amyloid precursor protein (*APP*) in AD pathogenesis (Tsatsanis et al. 2021) 2021). - The presence of *LTF* in the AD brain might be a counter-regulatory defence mechanism in which the elevation of *LTF* level may alleviate cognitive dysfunction and AD development(Bermejo‐Pareja et al. 2020; Mohamed et al. 2019; Reseco et al. 2021). |
| Alpha-2-HS-glycoprotein (*AHSG*) | - A serum biomarker of AD which serves as an anti-inflammatory protein that is suppressed by pro-inflammatory protein TNF-α, IL-1, and IL-6 (Geroldi et al. 2005; Smith et al. 2011). - The decrease in AHSG level in the brain was associated with cognitive failure in AD patients (Shi et al. 2019a; Shi et al. 2019b; Smith et al. 2011). |
| Coagulation factor IX (*F9*) | - The increase in the level of *F9* will be followed by cognitive decline in the AD brain (Begic et al. 2020). - Involves in the coagulation pathway that modulates the immune-inflammatory response in a series of cascade processes, which subsequently affects the development of neurodegeneration (De Luca et al. 2017; Festoff & Citron 2019; Suidan et al. 2018). |
| Selenoprotein P (*SELENOP)* | - A multifunctional protein that is essentially important to the human brain (Solovyev 2020; Zhang & Song 2021). - Inhibiting factor on Aβ and tau aggregation, suggesting the role of SELENOP in regulating AD development inversely (Du et al. 2014a; Du et al. 2014b; Rueli et al. 2017). |
| Retinol binding protein 4 (*RBP4)* | - Previously proposed as the target of therapeutic treatment for Late-onset AD (LOAD) (Goodman 2006). - Associated with type-II diabetes and obesity, which are among the validated risk factors for AD (Plucińska et al. 2016). |
| Extracellular matrix protein 1 (*ECM1)* | - Lower in abundance level of protein *ECM1* in plasma of AD compared to normal controls (François et al. 2022). - Showed downregulation activities in AD CSF model (Jiang et al. 2022). |
| Inter-alpha-trypsin inhibitor heavy chain H1 (*ITIH1*)*.* | - Relation with AD is unclear. - May transmit hyaluronan in plasma and involved in inflammatory pathways (Shen et al. 2017; Song et al. 2014). |
| Hepatocyte growth factor activator (*HGFAC*) | - An activator to hepatocyte growth factor (*HGF*), which is known to regulate certain brain functions including neuronal survival and development, in correlation with AD (Wright & Harding 2015; Zhu et al. 2018). - Although the increase in levels of *HGFAC* was reported to be accompanied by the elevation of *HGF* (Tsuboi et al., 2003), or it was identified as one of the significant variants in AD studies (Chen & Xia 2020; Prokopenko et al. 2021) the impact of *HGFAC* to AD development remains ambiguous. |
| Alpha-1B-glycoprotein (*A1BG*) | - Relation with AD is unclear. - Identified in a few AD-related studies as dysregulated protein (Khoonsari et al. 2019; Song et al. 2014; Srinivasan et al. 2020). |
| type II cytoskeletal 1 (*KRT1*) | - Relation with AD is unclear. |
| type I cytoskeletal 10 (*KRT10*) | - Relation with AD is unclear. |
