# Supplementary material for: A multi-ethnic proteomic profiling analysis in Alzheimer’s disease identifies the disparities in dysregulation of proteins and pathogenesis
Source: PeerJ. 2024 Jul 18;12:e17643. doi: 10.7717/peerj.17643 (PMC11260413; doi:10.7717/peerj.17643)
Supplement: Supplemental Information 5 [file peerj-12-17643-s005.docx]

Table S5: List of identified proteins in MADvC and their role and functions related to AD.

| Protein | Role and functions related to AD |
| --- | --- |
| Apolipoprotein A-IV (*APOA4*) | - Plays a crucial role in brain metabolism. - Functions as an anti-inflammatory agent (Cui et al. 2011; Vowinkel et al. 2004). - The decrement in the level of *APOA4* was observed with the increment of Aβ deposition level in the brain, resulting in cognitive damage (Cui et al. 2011; Lin et al. 2015). |
| Fibrinogen alpha chain (*FGA*) | - The accumulation of the *FGA* was detected in the AD brain in the study (Bian et al. 2021). - Triggers abnormal coagulation and fibrinolysis in the vascular unit in the brain, causing neurovascular inflammation and dysfunction (Shi et al. 2019a; Shi et al. 2019b). - Protein *FGA* was found inversely correlated with cognitive dysfunction in the Malaysian population (Rehiman et al. 2020). |
| Complement C2 (*C2*) | - *C2* is activated by Aβ and p-tau which take part in the cascade process of inducing inflammation (Shah et al. 2021; Tenner 2020). |
| Complement C4-A (*C4A*) | - Plasma level of *C4A* was observed to increase in AD (Bennett et al. 2012). |
| Inter-alpha-trypsin inhibitor heavy chain H4 (*ITIH4*) | - Responds to the acute-inflammatory phase of several pathology progress of several diseases including AD (Yang et al. 2012). - Higher expression of *ITIH4* in the AD mice model compared to the control, suggesting the potential of *ITIH4* as a marker for AD (Shi et al. 2019a; Shi et al. 2019b) . |
