# Supplementary material for: A multi-ethnic proteomic profiling analysis in Alzheimer’s disease identifies the disparities in dysregulation of proteins and pathogenesis
Source: PeerJ. 2024 Jul 18;12:e17643. doi: 10.7717/peerj.17643 (PMC11260413; doi:10.7717/peerj.17643)
Supplement: Supplemental Information 6 [file peerj-12-17643-s006.docx]

**Supplementary Methods**

Method details

Samples were analysed in accordance with APAF SOPs MS-096: Tandem Mass Tag (TMT) labelling, MS-100: S-Trap assisted proteolytic digestion, and MS-070: Q-Exactive Data Acquisition, as marked below wherever appropriate.

Instruments and related reagents

- Mass Spectrometer: Q-Exactive HF-X (Thermo Fisher Scientific)
- NanoLC system: UltiMate 3000 (Thermo Fisher Scientific)
- Peptide trap column: C18 PepMap 100, 5 µm, 100 Å, 300 µm × 5 mm (Thermo Fisher Scientific)
- Nano-LC column: Halo-C18, 160 Å, 2.7 µm, 100 µm x 30 cm
- Column temperature: 35 °C
- Loading buffer: 99.9% water, 0.1% formic acid
- Mobile phase A: 99.9% water, 0.1% formic acid
- Mobile phase B: 99.9% acetonitrile, 0.1% formic acid

Sample digestion

Protein samples were processed for mass spectrometric analysis using commercially procured S-Traps (Protifi, USA) (Provided by the client). Sample digestion procedures were performed as per manufacturer’s (Protifi, USA) instructions, in accordance with SOP MS-100. Briefly, sample were resuspended in S-Trap lysis buffer (46 µL, 5% SDS, 100 mM triethylammonium bicarbonate, TEAB, pH 7.55), sonicated in a water bath for 5 min and vortexed. Disulfide bonds were reduced with 10 mM DTT at 60 °C for 30 min, and then alkylated with 25 mM IAA for 30 min in dark at room temperature. Aliquots of 23.1 µL were acidified using 12 % aqueous phosphoric acid, added at 1:10 for a final concentration of ~1.2 % phosphoric acid and diluted using S-Trap binding buffer (90% aqueous methanol containing a final concentration of 100 mM TEAB, pH 7.55). S-Trap binding buffer was added to the acidified lysis buffer and the sample mixture was transferred to a labelled S-Trap column and centrifuged at 4,000 g, after which the flow through was discarded. The column was washed twice using S- Trap binding buffer and proteins retained on the column were digested in the presence of 125 µL trypsin solution (1:25 trypsin to protein ratio, total ~ 7.5 µg total trypsin in 50 mM triethylammonium bicarbonate) for 3 hours at 47 °C. Following digestion, peptides were eluted off the column after an addition of 50 mM triethylammonium bicarbonate and centrifugation. Remaining peptides were eluted from the column using a sequential centrifugation with addition of 0.2% aqueous formic acid followed by 50 % aqueous ACN containing 0.2 % formic acid. Peptides were dried by vacuum centrifugation then reconstituted in 200 mM HEPES (pH 8.8). Peptide concentration was determined using the Pierce quantitative colorimetric peptide assay (Thermo Scientific, USA).

Sample labelling

Single aliquots of equal peptide quantities from each of 6 samples (ADCF (APAF Sample ID: S0015897), ADMM (APAF Sample ID: S0015900), ADMF (APAF Sample ID: S0015899), CCF (APAF Sample ID: S0015901), (CFM APAF Sample ID: S0015903) and CCM (APAF Sample ID: S0015902)), and 2 of equal peptide quantities aliquots from each of ADCM (APAF Sample ID: S0015898) and CMM (APAF Sample ID: S0015904) were used for subsequent sample processing. Ten samples were labelled in a 10-plex TMT label batch as indicated in Table- 2. TMT reagent (Thermo Scientific, USA) labelling of each sample was performed as per APAF’s SOP MS-096.

Briefly, anhydrous acetonitrile was added to each TMT label vial followed by vortexing and brief centrifugation. Aliquots of individual peptide samples were labelled with one of the individual TMT labels (total of ten labels, Table-2). Labelling was performed at room temperature for 1 h with occasional vortexing. To quench the excess TMT label in the sample, 5 % hydroxylamine was added to each of the samples and vortexed briefly, then incubated at room temperature for 15 min. Before pooling the samples, a ‘label check’ experiment was performed to ensure equal amounts of total peptide were pooled from all samples. The label check was performed by mixing 2 µL aliquots of each individually labelled TMT sample, performing SDB-RPS StageTip clean-up and vacuum dried using a vacuum centrifuge. Label-check sample were reconstituted in 2 % ACN 0.1 % formic acid and analysed by LC-MS/MS following methods described below in Data acquisition. A normalization factor was obtained from the label check experiment and the original TMT-labelled peptide samples were then pooled at an equal, 1:1 ratio across all individual samples in the respective set.

| Label | Sample Code | Name in the result excel spreadsheet* |
| --- | --- | --- |
| 126 | ADCF | R1.126 |
| 127N | ADCM | R1.127N |
| 127C | ADMF | R1.127C |
| 128N | ADMM | R1. 128N |
| 128C | CCF | R1. 128C |
| 129N | CCM | R1.129N |
| 129C | CFM | R1.129C |
| 130N | CMM | R1. 130N |
| 130C | ADCM | R1.130C |
| 131 | CCM | R1.131 |

*Unique names assigned to each sample in the Excel work book of results enclosed with the report.

Offline Basic pH Reversed-phase (BpRP) Fractionation of TMT labelled peptides

The pooled peptides were dried in a vacuum centrifuge. The peptide mixture was resuspended in 900 µL of 0.1% TFA, then separated into 3 fractions using Pierce High pH reverse phase centrifugal columns. Aliquots of 300 µL were loaded to the column, and washed as per manufacturer’s instructions, before 3 fractions were eluted with 12.5 %, 17.5 % and 50 % acetonitrile in 0.1 % triethylamine, and fractions dried in a vacuum centrifuge. Samples were then reconstituted in 0.1 % formic acid for LC-MS/MS analysis.

Data dependent acquisition (DDA) LC-MS/MS

HpH fractionated TMT labelled peptides were subjected to LC-MS/MS analysis. Briefly, each peptide fraction was injected onto the peptide trap and washed with loading buffer for 10 minutes. The peptide trap was then switched in line with the analytical nano-LC column. Peptides were eluted from the trap onto the nano-LC column and peptides were separated with a linear gradient of 5% mobile phase B to 30% mobile over 110 min at a flow rate of 600 nL/min, followed by 85% B for 8 minutes.

The column eluent was directed into the ionization source of the mass spectrometer operating in positive ion mode. Peptide precursors from 350 to 1850 m/z were scanned at 60k resolution. The 10 most intense ions in the survey scan were fragmented by HCD using a normalized collision energy of 33 with a precursor isolation width of 0.8 m/z. Only precursors with charge state +2 to +5 were subjected to MS/MS analysis. The MS method had a minimum signal requirement value of 3000 ions for MS2 triggering, an AGC target value of 1×105 for MS2 and a maximum injection time of 85 ms. MS/MS scan resolution was set at 45,000 and dynamic exclusion was set to 30 seconds.

Protein identification and quantification

The raw data were processed using Proteome Discoverer (Version 2.1.0.81, Thermo Scientific). The data were searched using search engines SequestHT and Mascot against a sequence database for the *Homo sapiens*. The parameters for the data processing were attached as follows:

- Enzyme: Trypsin
- Maximum missed cleavages: 2
- Precursor mass tolerance: 20 ppm
- Fragment mass tolerance: 0.02 Da
- Dynamic modifications: Oxidation (M), Deamidated (N, Q), Glu->PyroGlu, Gln->Pyro-Glu, Acetyl (Protein N-Terminus), and TMT6plex (K) and TMT6plex (N-term)
- Static Modification: Carbamidomethyl (C)
- FDR and result in display filters: Protein, Peptide and PSM FDR<1%, Master proteins only
