# Supplementary material for: A multi-ethnic proteomic profiling analysis in Alzheimer’s disease identifies the disparities in dysregulation of proteins and pathogenesis
Source: PeerJ. 2024 Jul 18;12:e17643. doi: 10.7717/peerj.17643 (PMC11260413; doi:10.7717/peerj.17643)
Supplement: Supplemental Information 7 [file peerj-12-17643-s007.pdf]

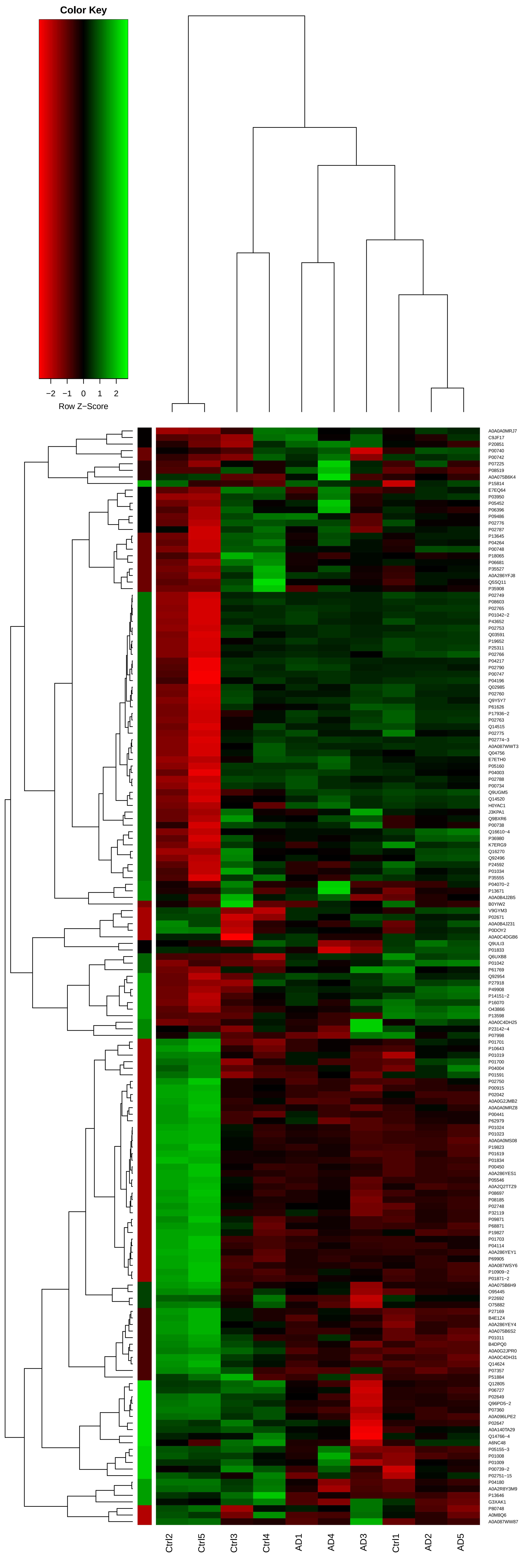
Color Key
−2 −1 0 1 2
Row Z−Score
A0A0A0MRJ7
C9JF17
P20851
P00740
P00742
P07225
P08519
A0A075B6K4
P15814
E7EQ64
P03950
P05452
P06396
P09486
P02776
P02787
P13645
P04264
P00748
P18065
P06681
P35527
A0A286YFJ8
Q5SQ11
P35908
P02749
P08603
P02765
P01042−2
P43652
P02753
Q03591
P19652
P25311
P02766
P04217
P02790
P00747
P04196
Q02985
P02760
Q9Y5Y7
P61626
P17936−2
P02763
Q14515
P02775
P02774−3
A0A087WWT3
Q04756
E7ETH0
P05160
P04003
P02788
P00734
Q9UGM5
Q14520
H0YAC1
J3KPA1
Q9BXR6
P00738
Q16610−4
P36980
K7ERG9
Q16270
Q92496
P24592
P01034
P35555
P04070−2
P13671
A0A0B4J2B5
B0YIW2
V9GYM3
P02671
A0A0B4J231
P0DOY2
A0A0C4DGB6
Q9ULI3
P01833
Q6UXB8
P01042
P61769
Q92954
P27918
P49908
P14151−2
P16070
O43866
P13598
A0A0C4DH25
P23142−4
P07998
P01701
P10643
P01019
P01700
P04004
P01591
P02750
P00915
P02042
A0A0G2JMB2
A0A0A0MRZ8
P00441
P62979
P01024
P01023
A0A0A0MS08
P19823
P01619
P01834
P00450
A0A286YES1
P05546
A0A2Q2TTZ9
P08697
P08185
P02748
P32119
P09871
P68871
P19827
P01703
P04114
A0A286YEY1
P69905
A0A087WSY6
P10909−2
P01871−2
A0A075B6H9
O95445
P22692
O75882
P27169
B4E1Z4
A0A286YEY4
A0A075B6S2
P01011
B4DPQ0
A0A0G2JPR0
A0A0C4DH31
Q14624
P07357
P51884
Q12805
P06727
P02649
Q96PD5−2
P07360
A0A096LPE2
P02647
A0A140TA29
Q14766−4
A6NC48
P05155−3
P01008
P01009
P00739−2
P02751−15
P04180
A0A2R8Y3M9
P13646
G3XAK1
P80748
A0M8Q6
A0A087WW87
Ctrl2
Ctrl5
Ctrl3
Ctrl4
AD1
AD4
AD3
Ctrl1
AD2
AD5
