# Supplementary material for: A multi-ethnic proteomic profiling analysis in Alzheimer’s disease identifies the disparities in dysregulation of proteins and pathogenesis
Source: PeerJ. 2024 Jul 18;12:e17643. doi: 10.7717/peerj.17643 (PMC11260413; doi:10.7717/peerj.17643)
Supplement: Supplemental Information 8 [file peerj-12-17643-s008.docx]

**Questionnaire to study demographic, physical activities and lifestyle impacts in Alzheimer’s Disease (AD) patients**

**Patient Information**

| Name |  |
| --- | --- |
| Age | < 47 years  47 – 53 years  >53 years |
| Gender | Male  Female |
| Race | Malay  Chinese  Indian  Others: |
| Nationality | Malaysian  Others: *Please specify* |
| Address |  |
| Living area | City centre  Town centre  Village  Isolated residency (e.g. farm, beach house, etc) |
| Marital status | Single  Married  Divorced  Widow |
| Living situation | Living alone  Living with spouse  Living with family members (e.g. children, relative, etc)  Others: *Please specify* |
| Highest education level | No formal  Primary  Secondary  Pre-university  Polytechnique/ University/ College  Others: *Please specify* |
| Highest certificate | None  UPSR  PMR  SPM/ IGCSE/ ‘O’ level/ equivalent  STPM/ Matriculation/ ‘A’ level/ equivalent  Diploma  Bachelor’s Degree  Master’s Degree  Doctorate  Professional |

**Physical Assessment**

| Height | cm | BMI | kg/m^2^ |
| --- | --- | --- | --- |
| Weight | kg | Systolic BP | mmHg |
| Waist | cm | Total Cholesterol | mmol/l |
| Hip | cm |  |  |

**Clinical Characteristic**

| Family history of Alzheimer’s Disease | Yes Please state the relation:  No |
| --- | --- |
| Medical conditions/ History | *(You may tick more than one)*  Diabetes mellitus  Hearing disease  Hypertension  Visual disease  Cerebrovascular disease  Cataract  Myocardial infarction  Gastrointestinal disease  Heart failure  Renal disease  Atrial fibrillation  Hepatitis  Peripheral vascular disease  Asthma  Epilepsy  Arthritis  Parkinsonism  Osteoporosis  Hyperthyroid  Others: Please state: _______________ |
| History of head injury | Yes  No |

**Physical Activities**

| **Part 1: Job Related Physical activity** | | |
| --- | --- | --- |
| 1. | Employment status | Employee  Self-Employment  Skip to Part2, Question 7 if:  Unemployed  Retired |
| 2. | Occupation |  |
| 3. | How many days do you involve physical activities (e.g. loads lifting, climbing upstairs, etc) for more than 10 mins in your work? | Never  1 to 3 times  4 to 5 times  More than 5 times |
| 4. | How many days do you walk for more than 10 mins (excluding travel to or from work) in your work? | Never  1 to 3 times  4 to 5 times  More than 5 times |
| **Part 2: Transportation Related Physical Activity** | | |
| 5. | What is your mode of the transportation for going work? | Public transport (e.g. train, bus, taxi)  Private transport (e.g. motorbike, car, etc)  Cycling  Walking |
| 6. | How much time do you usually spend on travelling for work in one time? | less than 10 minutes  10 to 20 minutes  20 to 30 minutes  more than 30 minutes |
| 7. | Other than going for work, how many days that you go outside of your house a week? | Never ---Skip to Part 3  2 to 3 days  4 to 5 days  Everyday |
| 8. | What is your mode of transportation (excluding the transportation you take to work)? *You may tick more than one option.* | Public transport (e.g. train, bus, taxi)  Private transport (e.g. motorbike, car, etc)  Cycling  Walking |
| 9. | How many days do you travel in a motor vehicle like train, bus or car in a week (excluding the transportation you take to work)? | Never  1 to 3 times  4 to 5 times  More than 5 times |
| 10. | On average, how much time do you spend on traveling in a motor vehicle like train, bus or car in one time (excluding the transportation you take to work)? | less than 10 minutes  10 to 30 minutes  30 minutes to 1 hour  more than 1 hour |
| 11. | Do you ride bicycle from place to place? | Yes  No ---Skip to Part2, Question 13 |
| 12. | How much time do you usually spend on cycle in one time (excluding the transportation you take to work)? | less than 10 minutes  10 to 20 minutes  20 to 30 minutes  more than 30 minutes |
| 13. | How much time do you usually spend on walking from place to place in one time (excluding the transportation you take to work)? | less than 10 minutes  10 to 20 minutes  20 to 30 minutes  more than 30 minutes |
| **Part 3: Housework, Recreation, Sport and Leisure-time Physical activity** | | |
| 14. | What kind of activity do you normally carry out when you are outside? | Dining  Exercise  House visit  Entertainment (e.g. watch movie, picnic, music concert, etc)  Run errands (e.g. groceries shopping, paying bills, dry cleaning, etc)  Doctor’s medical appointment |
| 15. | What kind of exercise that you normally participate in? | Light exercise (e.g. walking, housework, etc)  Moderate exercise (e.g. jogging, cycling, tai chi, gardening, etc)  Vigorous exercise (e.g. running, weight training, basketball, etc) |
| 16. | How much time do you usually spend on the exercise per day? | less than 10 minutes  10 to 30 minutes  more than 30 minutes |
| 17. | How much time do you spend sitting per day? | less than 1 hours  2 to 3 hours  more than 3 hours |

**Lifestyle and diet**

| 1. | How many hours do you sleep at night? | less than 5 hours  5 to 6 hours  6 to 7 hours  7 to 8 hours  more than 8 hours |
| --- | --- | --- |
| 2. | How many times do you wake up at night? | Never  1 to 2 times  3 times or more |
| 3. | Are you a smoker? | Current  Ex-smoker/ quit (less than a year ago)  Ex-smoker/ quit (more than a year)  Never smoke --Skip to Question 6 |
| 4. | How long have you been smoking? | less than 10 years  10 to 20 years  more than 20 years |
| 5. | How many cigarettes do you smoke per day? | 1 to 10  11 to 20  20+ |
| 6. | How often do you skip breakfast? | Never  Once to twice per week  More than twice per week |
| 7. | How often do you skip lunch? | Never  Once to twice per week  More than twice per week |
| 8. | How often do you skip dinner? | Never  Once to twice per week  More than twice per week |
| 9. | Do you eat snacks instead of proper meals on most days? | Never  Once to twice per week  More than twice per week |
| 10. | What style of eating habit do you have? | Vegetarian  Less vegetable / Mostly meat  Mostly carbohydrates (e.g. rice, noodle, etc)  Well balance (vegetables, meat, carbohydrates) |
| 11. | How many times a week do you eat fast food/ fried food/ food in high fat/salt? | Once or less  2 to 3 times  4 to 5 times  6 to 7 times  more than 7 times |
| 12. | How many sweeten drinks/ food (e.g. soda, dessert and etc) that you have in a week? | Once or less  2 to 3 times  4 to 5 times  6 to 7 times  more than 7 times |
| 13. | How many fruits do you consume per day? | Not at all  Once  2 to 3  More than 3 |
| 14. | Do you consume drinks containing alcohol? | Yes  No --- End of survey |
| 15. | How often do you drink alcoholic beverages? | Every day  4 to 6 times per week  1 to 3 times per week  On special occasions |
| 16. | How many drinks do you have each time?  *one unit= one pint/ one can of beer/ one small glass of wine/ one small glass of liquor | 1 to 3  4 to 6  7 to 9  more than 10 |

**THANK YOU FOR TAKING THIS SURVEY**
