# Supplementary material for: A multi-ethnic proteomic profiling analysis in Alzheimer’s disease identifies the disparities in dysregulation of proteins and pathogenesis
Source: PeerJ. 2024 Jul 18;12:e17643. doi: 10.7717/peerj.17643 (PMC11260413; doi:10.7717/peerj.17643)
Supplement: Supplemental Information 10 [file peerj-12-17643-s010.doc]

STROBE Statement—Checklist of items that should be included in reports of ***case-control studies***

|  | Item No | Recommendation |
| --- | --- | --- |
| **Title and abstract** | 1 | (*a*) Indicate the study’s design with a commonly used term in the title or the abstract (line 2-53) |
|  |  | (*b*) Provide in the abstract an informative and balanced summary of what was done and what was found (line 2-53) |
| Introduction | | |
| Background/rationale | 2 | Explain the scientific background and rationale for the investigation being reported (line 55-122) |
| Objectives | 3 | State specific objectives, including any prespecified hypotheses (line 108-122) |
| Methods | | |
| Study design | 4 | Present key elements of study design early in the paper (line 125, Figure 1) |
| Setting | 5 | Describe the setting, locations, and relevant dates, including periods of recruitment, exposure, follow-up, and data collection (line 127-141) |
| Participants | 6 | (*a*) Give the eligibility criteria, and the sources and methods of case ascertainment and control selection. Give the rationale for the choice of cases and controls (line 127-141) |
|  |  | (*b*) For matched studies, give matching criteria and the number of controls per case (line 127-141) |
| Variables | 7 | Clearly define all outcomes, exposures, predictors, potential confounders, and effect modifiers. Give diagnostic criteria, if applicable (line 127-141, 217-236) |
| Data sources/ measurement | 8* | For each variable of interest, give sources of data and details of methods of assessment (measurement). Describe comparability of assessment methods if there is more than one group (line 160-251) |
| Bias | 9 | Describe any efforts to address potential sources of bias (line 237-251) |
| Study size | 10 | Explain how the study size was arrived at (line 124-141) |
| Quantitative variables | 11 | Explain how quantitative variables were handled in the analyses. If applicable, describe which groupings were chosen and why (line 178-251) |
| Statistical methods | 12 | (*a*) Describe all statistical methods, including those used to control for confounding (line 217-236) |
|  |  | (*b*) Describe any methods used to examine subgroups and interactions (line 178-236) |
|  |  | (*c*) Explain how missing data were addressed (line 254-258) |
|  |  | (*d*) If applicable, explain how matching of cases and controls was addressed (N.A.) |
|  |  | (*e*) Describe any sensitivity analyses (N.A.) |
| Results | | |
| Participants | 13* | (a) Report numbers of individuals at each stage of study—eg numbers potentially eligible, examined for eligibility, confirmed eligible, included in the study, completing follow-up, and analysed (Table 1) |
|  |  | (b) Give reasons for non-participation at each stage (N.A.) |
|  |  | (c) Consider use of a flow diagram (Figure 1) |
| Descriptive data | 14* | (a) Give characteristics of study participants (eg demographic, clinical, social) and information on exposures and potential confounders (Table 1) |
|  |  | (b) Indicate number of participants with missing data for each variable of interest (N.A.) |
| Outcome data | 15* | Report numbers in each exposure category, or summary measures of exposure (line 269-316) |
| Main results | 16 | (*a*) Give unadjusted estimates and, if applicable, confounder-adjusted estimates and their precision (eg, 95% confidence interval). Make clear which confounders were adjusted for and why they were included (line 253-356) |
|  |  | (*b*) Report category boundaries when continuous variables were categorized (N.A.) |
|  |  | (*c*) If relevant, consider translating estimates of relative risk into absolute risk for a meaningful time period (N.A.) |

| Other analyses | 17 | Report other analyses done—eg analyses of subgroups and interactions, and sensitivity analyses  (line 317-356) |
| --- | --- | --- |
| Discussion | | |
| Key results | 18 | Summarise key results with reference to study objectives (line 359-478) |
| Limitations | 19 | Discuss limitations of the study, taking into account sources of potential bias or imprecision. Discuss both direction and magnitude of any potential bias (line 480-507) |
| Interpretation | 20 | Give a cautious overall interpretation of results considering objectives, limitations, multiplicity of analyses, results from similar studies, and other relevant evidence (line 509-519) |
| Generalisability | 21 | Discuss the generalisability (external validity) of the study results (line 522-544) |
| Other information | | |
| Funding | 22 | Give the source of funding and the role of the funders for the present study and, if applicable, for the original study on which the present article is based (This work was supported by the Fundamental Research Grant Scheme (FRGS), Ministry of Higher Education Malaysia, project number FRGS/1/2019/SKK06/UM/02/5 and UM International Collaboration Grant, project number ST041-2022. The funders had no role in the study, design, data collection and analysis, the decision to publish, or the preparation of the manuscript) |

*Give information separately for cases and controls.

**Note:** An Explanation and Elaboration article discusses each checklist item and gives methodological background and published examples of transparent reporting. The STROBE checklist is best used in conjunction with this article (freely available on the Web sites of PLoS Medicine at http://www.plosmedicine.org/, Annals of Internal Medicine at http://www.annals.org/, and Epidemiology at http://www.epidem.com/). Information on the STROBE Initiative is available at http://www.strobe-statement.org.
